# Stereoselective Analysis of the Antiseizure Activity of Fenfluramine and Norfenfluramine in Mice: Is *l*-Norfenfluramine a Better Follow-Up Compound to Racemic-Fenfluramine?

**DOI:** 10.3390/ijms25052522

**Published:** 2024-02-21

**Authors:** Natalia Erenburg, Emilio Perucca, Jeff Bechard, Celine Dube, Nina Weishaupt, Robin Sherrington, Meir Bialer

**Affiliations:** 1 Institute of Drug Research, School of Pharmacy, Faculty of Medicine, The Hebrew University of Jerusalem, Jerusalem 9112002, Israel; 2 Department of Medicine (Austin Health), University of Melbourne, Heidelberg, VIC 3084, Australia; 3 Department of Neuroscience, Central Clinical School, Monash University, Melbourne, VIC 3168, Australia; 4 Xenon Pharmaceuticals Inc., 3650 Gilmore Way, Burnaby, BC V5G 4W8, Canada; 5 David R. Bloom Center for Pharmacy, The Hebrew University of Jerusalem, Jerusalem 9112002, Israel

**Keywords:** fenfluramine, norfenfluramine, enantioselectivity, chiral switch, anticonvulsant activity, pharmacokinetic-pharmacodynamic correlation

## Abstract

The aim of this study was to investigate the comparative antiseizure activity of the *l*-enantiomers of *d*,*l*-fenfluramine and *d*,*l*-norfenfluramine and to evaluate the relationship between their concentration in plasma and brain and anticonvulsant activity. *d*,*l*-Fenfluramine, *d*,*l*-norfenfluramine and their individual enantiomers were evaluated in the mouse maximal electroshock seizure (MES) test. *d*,*l*-Fenfluramine, *d*,*l*-norfenfluramine and their individual *l*-enantiomers were also assessed in the DBA/2 mouse audiogenic seizure model. All compounds were administered intraperitoneally. Brain and plasma concentrations of the test compounds in DBA/2 mice were quantified and correlated with anticonvulsant activity. In the MES test, fenfluramine, norfenfluramine and their enantiomers showed comparable anticonvulsant activity, with ED_50_ values between 5.1 and 14.8 mg/kg. In the audiogenic seizure model, *l*-norfenfluramine was 9 times more potent than *d*,*l*-fenfluramine and 15 times more potent than *l*-fenfluramine based on ED_50_ (1.2 vs. 10.2 and 17.7 mg/kg, respectively). Brain concentrations of all compounds were about 20-fold higher than in plasma. Based on brain EC_50_ values, *l*-norfenfluramine was 7 times more potent than *d*,*l*-fenfluramine and 13 times more potent than *l*-fenfluramine (1940 vs. 13,200 and 25,400 ng/g, respectively). EC_50_ values for metabolically formed *d*,*l*-norfenfluramine and *l*-norfenfluramine were similar to brain EC_50_ values of the same compounds administered as such, suggesting that, in the audiogenic seizure model, the metabolites were responsible for the antiseizure activity of the parent compounds. Because of the evidence linking *d*-norfenfluramine to *d*,*l*-fenfluramine to cardiovascular and metabolic adverse effects, their *l*-enantiomers could potentially be safer follow-up compounds to *d*,*l*-fenfluramine. We found that, in the models tested, the activity of *l*-fenfluramine and *l*-norfenfluramine was comparable to that of the corresponding racemates. Based on the results in DBA/2 mice and other considerations, *l*-norfenfluramine appears to be a particularly attractive candidate for further evaluation as a novel, enantiomerically pure antiseizure medication.

## 1. Introduction

**Figure 1:**
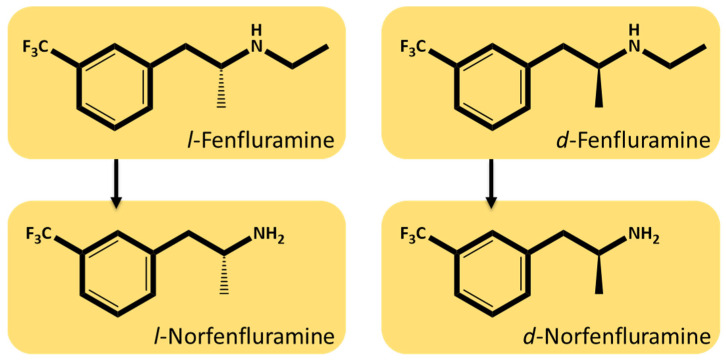
Chemical structures of the two individual enantiomers of fenfluramine and its primary active metabolite norfenfluramine.

An oral aqueous solution (Fintepla^®^, UCB, Brussels, Belgium) of *d*,*l*-fenfluramine HCl (referred to hereafter as fenfluramine) was approved in 2020 in the US and Europe for the treatment of seizures associated with Dravet syndrome and Lennox-Gastaut syndrome in patients aged 2 years and older [1,2]. Administration of fenfluramine results in four active chiral chemical entities being present in the systemic circulation, namely *d*- and *l*-fenfluramine and *d*- and *l*-norfenfluramine (Figure 1) [3]. Although differences in the pharmacokinetics and pharmacodynamics of the individual enantiomers of fenfluramine and its primary active metabolite *d*,*l*-norfenfluramine (referred to hereafter as norfenfluramine) have been known for decades [4,5,6,7,8,9,10], it is remarkable that recent articles on fenfluramine’s clinical pharmacology, mechanisms of action and drug interactions consider the medication as if it were a single molecular entity [11,12,13,14,15].

Fenfluramine and its *d*-enantiomer had been marketed in the past as appetite suppressants, and both were withdrawn from the market in 1997 after being found to cause valvular heart disease and pulmonary hypertension [3]. Although, to date, no cardiovascular toxicity has been reported in patients with epilepsy treated with fenfluramine, the drug is currently available in the US and Europe via a controlled access program requiring echocardiogram assessments before, during and after treatment [1,2].

Available evidence indicates that appetite suppression and cardiovascular toxicity are mediated by different serotonergic mechanisms. In particular, appetite suppression can be ascribed mainly to the *d*-enantiomers of fenfluramine and norfenfluramine [5,9], whereas cardiovascular toxicity can be ascribed mainly to the activation of 5-HT_2B_ receptors by *d*-norfenfluramine [5,7,16,17,18,19]. *d*-Norfenfluramine and *l*-norfenfluramine are also the major circulating metabolites of the fenfluramine analogue benfluorex ((±)-N-(2-benzoyloxyethy1)-norfenfluramine, Mediator^®^), which was approved in France as an adjuvant antidiabetic and withdrawn in 2009 due to its association with valvular heart disease and pulmonary hypertension [3,18,19,20].

With respect to antiseizure activity in preclinical models, fenfluramine has been found to protect against maximal electroshock seizures (MES) and audiogenic seizures in rodents [21,22,23]. It has also been shown to inhibit seizure-related locomotor activity and brain epileptiform discharges in zebrafish models of Dravet syndrome [24]. We recently reported the results of a comparative assessment of the antiseizure activity of the individual enantiomers of fenfluramine and norfenfluramine after intraperitoneal (i.p.) administration in rats [25]. Median effective doses (ED_50_) in the MES test were 8.4 mg/kg for *d*-fenfluramine, 13.4 mg/kg for *l*-fenfluramine and 10.2 mg/kg for *l*-norfenfluramine. A median effective dose for *d*-norfenfluramine could not be determined due to dose-limiting neurotoxicity. Because of the evidence linking *d*-fenfluramine and *d*-norfenfluramine to cardiovascular toxicity, these findings justify interest in developing *l*-fenfluramine or *l*-norfenfluramine as a potential, enantiomerically pure follow-up compound to the marketed fenfluramine [26,27].

The objective of the present study was to assess the comparative anticonvulsant activity of fenfluramine, norfenfluramine and their individual enantiomers in the MES test in mice. Additional experiments were conducted to assess the antiseizure effects of fenfluramine, norfenfluramine and their individual *l*-enantiomers in the DBA/2 mouse model of audiogenic seizures and their correlation with the concentration of the same compounds in plasma and brain. Together with the results of our previous pharmacodynamics/pharmacokinetic analysis in rats [25,28], these data will assist in determining whether *l*-fenfluramine or *l*-norfenfluramine is a better candidate compound for further development as a follow-up to the marketed racemic-fenfluramine.

## 2. Results

### 2.1. Antiseizure Activity and Protective Index in the MES Model in Mice

Fenfluramine, norfenfluramine and their individual enantiomers were active in protecting mice against electrically induced seizures at doses ranging from 4 to 30 mg/kg. Median effective doses (ED_50_) were assessed at the time of peak seizure protection, which occurred at 6 h after dosing for all compounds with the exception of *l*-fenfluramine and *l*-norfenfluramine, for which the time to peak seizure protection was 4 h and 8 h, respectively. By contrast, peak toxicity in the rotarod test developed early after dosing and median toxic doses (TD_50_) were assessed at 0.25 h after dosing for all compounds. 

Results of the experiments conducted to assess ED_50_ and TD_50_ are summarized in Table 1, whereas calculated ED_50_ and TD_50_ values with corresponding 95%CI and PI values are shown in Table 2. Fenfluramine, *d*-fenfluramine and *l*-fenfluramine were equipotent in terms of seizure protection, but the *l*-enantiomer was better tolerated and consequently had a higher PI. With respect to norfenfluramine, the *d*-enantiomer was more potent than the *l*-enantiomer but also more toxic, and the PI tended to favour *l*-norfenfluramine.

For comparison purposes, Table 2 also shows results in the MES test for the same compounds in rats, based on a previous publication [25]. In rats, all enantiomers had similar ED_50_ values except for *d*-norfenfluramine, which was more potent but also more toxic.

### 2.2. Studies in the DBA/2 Mouse Model of Audiogenic Seizures

**Figure 2:**
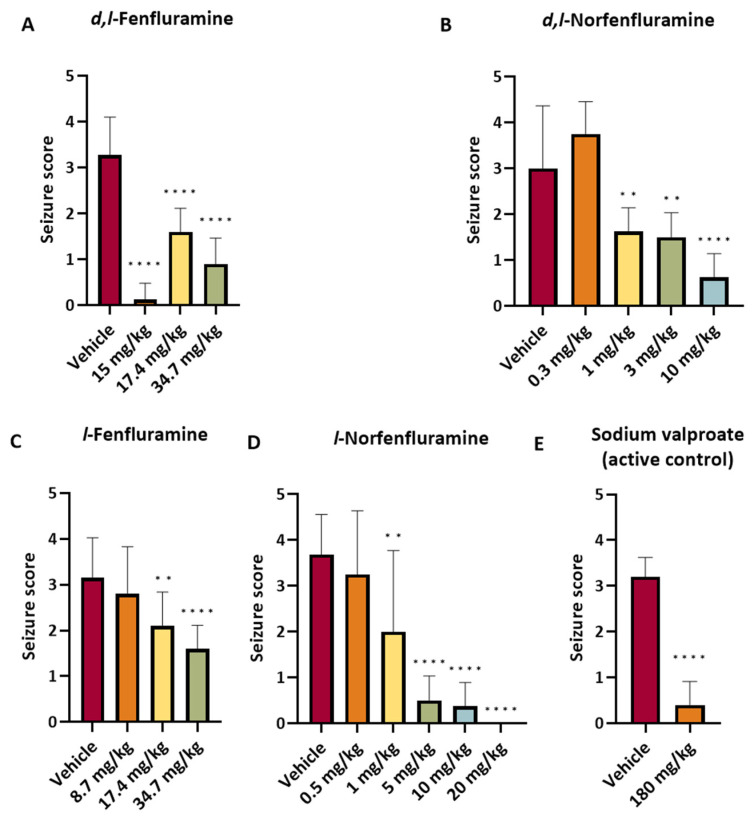
Relationship between dose and antiseizure response to *d*,*l*-fenfluramine (**A**), *d*,*l*-norfenfluramine (**B**) and their *l*-enantiomers (**C**,**D**) in the DBA/2 mouse model of audiogenic seizures. All doses are expressed as the HCl salt. Sodium valproate (180 mg/kg) was included as positive control (**E**). The antiseizure response was assessed at 1 h after a single i.p. administration, except for sodium valproate, which was assessed at 0.5 h after a single i.p. administration. Bars show mean ± SD. ** *p* < 0.01; **** *p* < 0.0001 (vs. vehicle). Dose-group comparisons were analyzed using one-way ANOVA followed by Dunnett’s multiple comparisons test (comparing each dose group with a vehicle).

All compounds tested caused dose-dependent protection against audiogenic seizures (Table 3 and Figure 2). ED_50_ values of norfenfluramine and *l*-norfenfluramine were almost identical (1.3 and 1.2 mg/kg, respectively) and greater than the ED_50_ values of fenfluramine and *l*-fenfluramine, respectively (10.2 and 17.7 mg/kg, respectively) (Table 4). Of note, *l*-norfenfluramine at the dose of 20 mg/kg was the only compound that fully suppressed seizures in all eight animals tested. The active control sodium valproate (180 mg/kg) exerted a degree of seizure protection similar to that observed with 0.5 and 10 mg/kg *l*-norfenfluramine and 10 mg/kg norfenfluramine (Figure 2).

**Figure 3:**
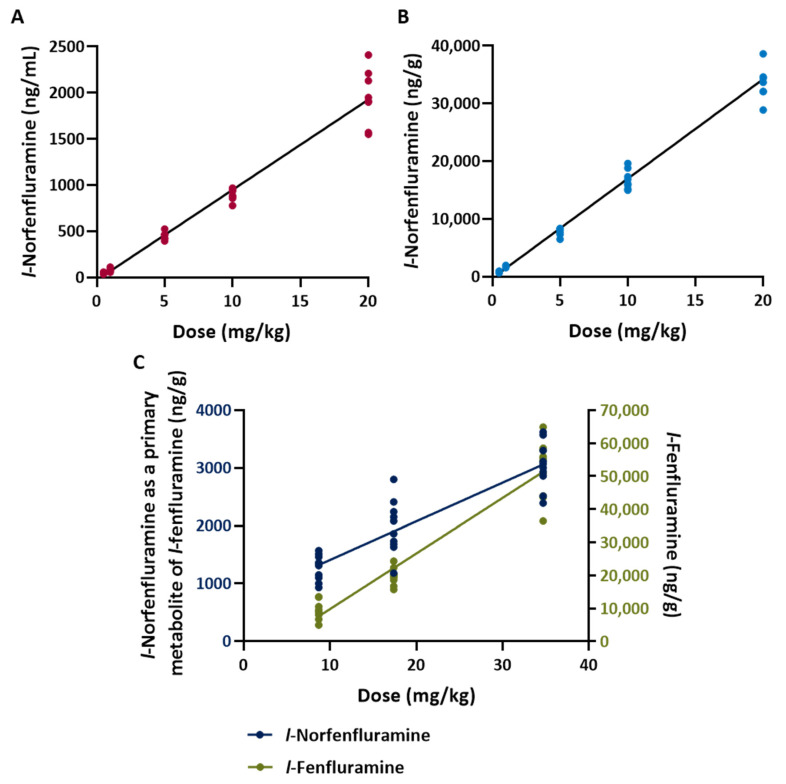
Relationship between *l*-norfenfluramine concentration in plasma (**A**) and brain (**B**) and dose in DBA/2 mice. Samples were collected immediately after completion of sound stimulation testing, which was conducted at 1 h following single i.p. doses of the compound. The figure also shows the relationship (**C**) between the brain concentration of *l*-fenfluramine and metabolically formed *l*-norfenfluramine after single i.p. doses of *l*-fenfluramine. All doses are expressed as the HCl salt.

The concentrations of each of the compounds tested (including the concentrations of metabolically formed norfenfluramine and *l*-norfenfluramine in animals treated with fenfluramine and its *l*-enantiomer, respectively) measured in brain samples collected soon after completion of audiogenic seizure testing are reported in Table 3. Irrespective of the dose administered, fenfluramine, norfenfluramine and *l*-norfenfluramine were found in brain tissue at concentrations about 20-fold higher than in plasma (Table 3). After administration of fenfluramine and *l*-fenfluramine, the concentration of the metabolically formed norfenfluramine and *l*-norfenfluramine in the brain was about 20-fold higher than the brain concentration of the parent compound (Table 3). For all compounds tested, brain concentrations appeared to be linearly related to dose (Table 3 and Figure 3).

**Figure 4:**
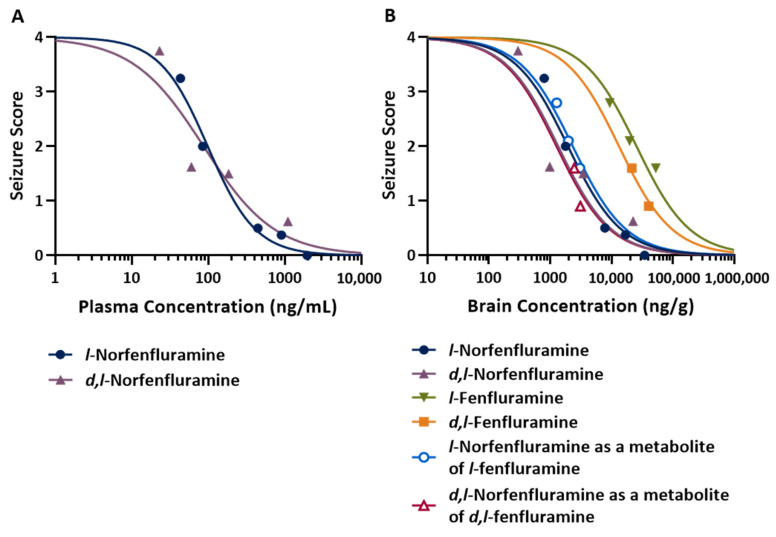
(**A**) Plasma concentration-response curve of *d*,*l*-norfenfluramine and *l*-norfenfluramine in the DBA/2 mouse model of audiogenic seizures. (**B**) Brain concentration-response curve of *d*,*l*-fenfluramine, *l*-fenfluramine, *d*,*l*-norfenfluramine and *l*-norfenfluramine in the same model. In B, brain concentration-response curves are also shown for *d*,*l*-norfenfluramine and *l*-norfenfluramine formed metabolically after i.p. administration of *d*,*l*-fenfluramine and *l*-fenfluramine, respectively. Plasma and brain samples were collected immediately after completion of sound stimulation testing, which was conducted at 1 h after dosing. For both plasma and brain, each data point represents the average of concentrations measured in 8–10 mice per dose group. Median effective concentrations (EC_50_) calculated from these curves are shown in Table 3. In (**A**), the Hill coefficient or steepness of concentration-response relationship (n) for *l*-and *d*,*l*- norfenfluramine was −1.388 and −0.9352, respectively. In (**B**), n was constrained to −1.000 in the analysis of concentration-response curves of all compounds.

Assessment of the concentration-response relationship (Figure 4) allowed the calculation of median effective concentrations (EC_50_) in both plasma and brain tissue. Because plasma samples were not collected in dose–response experiments with fenfluramine and *l*-norfenfluramine, plasma EC_50_ values could be determined only for norfenfluramine and its *l*-enantiomer and were found to be similar for both compounds (81 and 101 ng/mL, respectively), and much lower than their EC_50_ in the brain (1340 and 2330 ng/mg, respectively). The antiseizure potencies of norfenfluramine and *l*-norfenfluramine as estimated by EC_50_ values in the brain were 10- and 13-fold higher than those of fenfluramine and *l*-fenfluramine, respectively (Table 4). Of note, brain EC_50_ values for metabolically formed norfenfluramine and *l*-norfenfluramine were very similar to brain EC_50_ values of the same compounds administered as such (Table 4).

## 3. Discussion

Clarification of potential differences in antiseizure activity and safety profile of the individual enantiomers of fenfluramine and norfenfluramine has important implications because it could provide the rationale for developing a single enantiomer as a novel molecular entity with a better therapeutic index than the marketed racemic-fenfluramine. The evidence linking *d*-fenfluramine and *d*-norfenfluramine to cardiovascular adverse effects (and the withdrawal of *d*-fenfluramine from the market due to these complications) [5,7,16,17,18] may justify a chiral switch approach [26,27,29] aimed at developing *l*-fenfluramine or *l*-norfenfluramine as safer alternatives to fenfluramine. Assessing the comparative antiseizure activity of fenfluramine, norfenfluramine, and their *l*-enantiomers is a key step in determining the viability of this approach.

To date, few studies have investigated the antiseizure effects of *l*-fenfluramine and *l*-norfenfluramine in seizure models. In the MES test in rats, all enantiomers of fenfluramine and norfenfluramine have been found to be active in protecting against seizures, with a potency comparable to that of the corresponding racemates [25]. Our results show that in the MES test in mice, ED_50_ values of individual enantiomers are similar to those obtained previously in rats, including a trend for *d*-norfenfluramine to be more potent but also more toxic. Both in rats and in mice, ED_50_ values, which differed between species, were assessed at the time of peak seizure protection. In rats, peak antiseizure effects occurred at 15 to 30 min after dosing (except for *d*-fenfluramine with a peak effect at 2 h) [25], whereas in mice, the peak effect was observed at 4-8 h after dosing. In contrast, the peak effect for neurotoxic manifestations (MMI) was shorter and occurred between 15–30 min in both species.

A limitation of the MES studies summarized above is that no measurements were made of plasma and brain concentrations of the test compounds at the time at which response was assessed. Our subsequent experiments were conducted to compare *l*-fenfluramine and *l*-norfenfluramine with the corresponding racemic compounds in the DBA/2 mouse model of audiogenic seizures, assessed not only dose–response relationships but also concentration-response relationships. The DBA/2 model was selected for these experiments because this model permits a quick and reliable assessment of the antiseizure activity of drug candidates [23] and because racemic fenfluramine had previously been found to be active in a closely related model of audiogenic seizures [30]. ED_50_ values for *l*-norfenfluramine and norfenfluramine in the DBA/2 mouse model were almost identical (1.2 and 1.3 mg/kg). Based on ED_50_, *l*-norfenfluramine and norfenfluramine were 15 and 8 times more potent, respectively, than *l*-fenfluramine and fenfluramine. This finding is interesting because those compounds showed similar anticonvulsant potency in the MES model. A brain-to-plasma concentration ratio close to 20 for fenfluramine, norfenfluramine and their *l*-enantiomers is similar to the brain-to-plasma exposure (AUC) ratio previously reported for the same compounds in rats [25], indicating rapid and extensive brain penetration in both species. Based on brain EC_50_ values, norfenfluramine is 10 times more potent than fenfluramine, and *l*-norfenfluramine is 13 times more potent than *l*-fenfluramine in protecting against audiogenic seizures. The fact that the brain EC_50_ of *l*-norfenfluramine administered as such (1940 ng/g) was similar to that of metabolically formed *l*-norfenfluramine after administration of *l*-fenfluramine (2330 ng/g) strongly suggests that the antiseizure activity of *l*-fenfluramine in this model can be mainly ascribed to its primary metabolite. Likewise, the metabolite norfenfluramine appears to be the main determinant of the antiseizure effect observed after administration of fenfluramine. Of note, the plasma EC_50_ of *d*,*l*-norfenfluramine in the DBA/2 audiogenic seizure model (81 ng/mL) is well within the plasma concentration range (2.6–149.6 ng/mL) reported in patients with Dravet syndrome and other developmental and epileptic encephalopathies treated with fenfluramine in routine clinical practice [11].

Overall, the findings in the MES model in rats and mice and the audiogenic seizure model in DBA/2 mice provide clear evidence that both *l*-fenfluramine and *l*-norfenfluramine possess antiseizure activity and, therefore, support the rationale for their clinical development as enantiomerically pure ASMs. While the MES studies did not permit direct assessment of the relative contribution of metabolically formed norfenfluramine and *l*-norfenfluramine to the activity of fenfluramine and *l*-fenfluramine, the findings in DBA/2 mice provide strong evidence that the metabolite was responsible for the seizure protection observed after administration of the parent compound. The results in DBA/2 mice support the development of *l*-norfenfluramine, preferably *l*-fenfluramine, although it is unclear to what extent the same findings can be extrapolated to other models or to the clinical setting. While the MES test is generally considered predictive of activity against generalized tonic-clonic seizures and focal-to-bilateral tonic-clonic seizures [31], the specificity of the audiogenic seizure model in predicting the spectrum of activity against different seizure types is less clearly defined [30]. Both enantiomers of fenfluramine and norfenfluramine have been found to be inactive in the 6 Hz seizure model in mice [25]. Only one study evaluated fenfluramine and norfenfluramine enantiomers in a model designed to mimic Dravet syndrome. In the zebrafish *scn1Lab^−^*^/*−*^ mutant model of Dravet syndrome, all fenfluramine and norfenfluramine enantiomers were found to be similarly effective in inhibiting behavioural (locomotor) epileptic activity, but at the concentrations tested (up to 100 μM) *l*-norfenfluramine differed from the other enantiomers in failing to reduce the frequency and duration of epileptiform events in local field potential recordings [24]. In view of these findings, studies on the comparative activity of *l*-fenfluramine and *l*-norfenfluramine in other seizure models would be desirable.

There is sound evidence linking the cardiovascular adverse effects of fenfluramine to stimulation of 5-HT_2B_ receptors [5,7,16,17,18]. While fenfluramine per se does not bind significantly to 5-HT_2B_ receptors, *d*-norfenfluramine activates these receptors with at least 10-fold greater potency compared with *l*-norfenfluramine [16,32,33,34]. In addition to minimizing the risk of cardiovascular adverse effects, *l*-norfenfluramine (or *l*-fenfluramine), as a safer follow-up compound to fenfluramine, could have additional advantages. In particular, the appetite-suppressant effects of fenfluramine are known to be mediated at least in part by activation of 5-HT_2c_ receptors by the metabolite norfenfluramine [3,35]. Because *l*-norfenfluramine is at least 3-times less potent than *d*-norfenfluramine in activating this receptor [16], it would be expected to have a lower potential to induce anorexia and weight loss.

As an indication of the interest in developing individual enantiomers as follow-up compounds to fenfluramine, at least two companies have filed patent applications for the use of the enantiomers of fenfluramine and norfenfluramine as antiseizure agents [3]. On 28 June 2023, the European Patent Office issued a Notice of Intent to grant a patent on the use of *l*-norfenfluramine in epilepsy [36]. The comparative antiseizure activity of *l*-norfenfluramine and *l*-fenfluramine has been discussed above. In addition to the data in the audiogenic seizure model, arguments favouring the preferential development of *l*-norfenfluramine over *l*-fenfluramine include the advantage of a single active molecular entity, a longer half-life consistent with once-daily dosing [6], and a lower susceptibility to enzyme inhibition- or induction-based drug–drug interactions [12]. Compared to *l*-fenfluramine, *l*-norfenfluramine would also be expected to have a lower pharmacokinetic variability due to minimal cytochrome P450 (CYP)-mediated metabolism and avoidance of the CYP2D6 genetic polymorphism affecting fenfluramine’s disposition [37].

## 4. Materials and Methods

### 4.1. Materials

Fenfluramine HCl (lot no. 10-JKL-74-1), *d*-fenfluramine HCl (lot no. 10-DHL-18-1) and *l*-fenfluramine HCl (lot no. 10-DHL-26-1) were obtained from Toronto Research Chemicals (North York, ON, Canada). Norfenfluramine HCl (lot no. CD-890-72) *d*-norfenfluramine HCl (lot no. MRA-879-77) and *l*-norfenfluramine HCl (lot no. TC-055-208 and TC-150-097) were obtained from BioVectra (Charlottetown, PE, Canada) and sodium valproate (lot n. MKCD3350) from Sigma Aldrich (Oakville, ON, Canada). Mice for the MES studies were purchased from Charles River (Kingston, NY, USA). DBA/2 mice were obtained from Charles River (Kingston, NY, USA) for studies in Canada and from Janvier Laboratories (Le Genest-Saint-Isle, France) for studies in France.

All doses of fenfluramine, norfenfluramine and their individual enantiomers are expressed as the HCl salt. All concentrations are expressed as free bases.

### 4.2. Assessment of Antiseizure Activity and Neurotoxicity in the Mouse MES Model

All experiments were conducted in male albino CF-1/Charles River mice (18–30 g) as part of the NIH Epilepsy Therapy Screening Program (ETSP) at the ETSP contract site at the University of Utah (Salt Lake City, UT, USA) [38,39,40]. Investigators at the testing site were blinded to the source and identity of all compounds tested. All protocols involving the use of animals were approved by the Institutional Animal Care and Use Committee at the University of Utah.

Test compounds were administered, i.p. Details of the experimental procedures have been described before [25]. The results of initial exploratory experiments conducted to assess the time course of antiseizure activity and neurotoxic effects have also been reported previously [25]. Median effective dose (ED_50_) and median minimal neurotoxic dose (TD_50_) values with 95% confidence intervals (95%CI) were determined by testing 4 to 5 doses, each in 8 animals, at the time of corresponding peak effects. For the assessment of antiseizure activity, the electric stimulus (0.2 s, 60 Hz AC, 50 mA) was delivered using corneal electrodes. Seizure protection was defined as the abolition of the hindlimb tonic extensor component of the seizure. The determination of TD_50_ was based on an assessment of minimal motor impairment (MMI) in the rotarod test. In this test, the animal is placed on a rotating rod (6 rpm), and MMI is considered to be present if the animal falls from the rod three times during the 1 min testing period. Any abnormal behaviour manifestations were also recorded. The protective index (PI) was calculated as the ratio between TD_50_ and ED_50_ values.

### 4.3. Assessment of Antiseizure Activity in the DBA/2 Mouse Model of Audiogenic Seizures

The audiogenic seizure assay was conducted at 1 h after dosing, except for sodium valproate (active control), which was assessed at 0.5 h after dosing. All compounds were administered i.p. and dissolved in 0.9% sodium chloride in water. The volume injected was 10 mL/kg for all compounds and doses. The study was conducted at two sites, Xenon Pharmaceuticals Inc. (Vancouver, BC, Canada) and Porsolt (Le Genest-Saint-Isle, France). The two sites evaluated different treatments using virtually identical experimental protocols. Protocols involving the use of animals at Xenon Pharmaceuticals Inc. were approved by the Xenon Animal Care Committee and followed the guidelines of the Canadian Council on Animal Care (CCAC). Studies performed at Porsolt were done at an Association for Assessment and Accreditation of Laboratory Animal Care International (AAALAC)-accredited facility.

The Canadian site tested *l*-norfenfluramine (0.5–20 mg/kg), norfenfluramine (0.3–5 mg/kg) and fenfluramine (15 mg/kg). Male DBA/2 mice (7.6–18.5 g, 3–4 weeks old) were individually placed in a clear acrylic cubic box with a lid (40.6 cm on each side) and allowed to explore the box freely for 1 min. After this period of habituation, an auditory stimulus was generated using an electrical bell at an intensity of about 105 dB for a maximum duration of 60 s or until mice exhibited a tonic seizure. 

The French site tested *l*-fenfluramine (8.7–34.7 mg/kg), mg/kg), fenfluramine (17.4 and 34.7 mg/kg) and sodium valproate (180 mg/kg). Male DBA/2 mice (5–10 g, 3–4 weeks old) were placed in a Plexiglas jar (diameter 40 cm; height 35 cm) mounted with an electric bell (105–120 dB). The bell was activated for a maximum duration of 60 s unless death occurred earlier.

At both sites, seizure-related behaviour after sound stimulation was evaluated for 10 min according to the method described by Dürmüller et al. 1993 [41]. Specifically, the score was 0 for no seizure, 1 for wild running, 2 for clonic convulsion, 3 for tonic extension, and 4 for death. Soon after completion of the experiment, samples of blood and brain were obtained for pharmacokinetic analysis (see below). Experimenters were blind to the treatment conditions, except for sodium valproate (dosed 30 min prior to seizure induction).

### 4.4. Assay of Fenfluramine and Norfenfluramine in Biological Samples

#### 4.4.1. Sample Collection and Preparation

At the Canadian site, DBA/2 mice were anesthetized using isoflurane inhalation soon after the completion of the antiseizure activity testing. A 0.5 mL of blood was collected from the heart, deposited into a K_2_EDTA tube and stored on ice. Animals were then euthanized using cervical dislocation, and their brains were removed, placed into pre-weighed vials, and snap-frozen on dry ice. At the end of the sample collection, blood was centrifuged at 4000 rpm for 10 min at 4 °C, and the plasma was pipetted into a labelled tube. All samples were stored in a freezer at −80 °C until bioanalysis.

At the French site, animals were decapitated at the completion of each experiment. The brain was quickly removed and rinsed with physiological saline. The whole brain and individual hemispheres were weighed, placed in separated pre-labelled vials and snap-frozen in liquid nitrogen (or equivalent). The vials were stored at −80 °C until shipment on dry ice to Xenon Pharmaceuticals for the measurement of fenfluramine, *l*-fenfluramine, norfenfluramine and *l*-norfenfluramine concentrations.

#### 4.4.2. Quantification of Norfenfluramine and L-norfenfluramine in Plasma

Extraction of plasma samples was carried out by protein precipitation using acetonitrile. Fifty microliters of plasma sample were mixed with 50 μL of internal standard solution in 1:1 acetonitrile: water (*v*/*v*) followed by the addition of 50 μL of 6% (*v*/*v*) phosphoric acid in water and 200 μL of acetonitrile. The mass of the internal standard and mass fragments used for detection as a transition was 203.96/159 *m*/*z*. Samples were vortexed for 30 s, then centrifuged at 13,000 rpm for 20 min. Fifty microliter aliquots of each supernatant were added into a 96-well plate, followed by the addition of 1:1 acetonitrile: water (*v*/*v*) to bring the final volume to 250 μL in the well plate. After mixing, the plate was further centrifuged at 4000 rpm for 20 min. The samples were analyzed using ultra-high pressure liquid chromatography-electrospray ionization tandem mass spectrometry (UHPLC-ESI-MS/MS) using a Sciex TQ-5500 triple quadrupole mass spectrometer (Framingham, MA, USA) equipped with a Shimadzu Nexera UHPLC (Columbia, MD, USA) pump and auto-sampler system using an ACE Excel C18-PFP, 50 × 2.1 mm, 2 μm particle size column and gradient elution consisting of solvent A (0.1% formic acid in water) and solvent B (0.1% formic acid in acetonitrile). The analytes (norfenfluramine and its *l*-enantiomer) were detected using electrospray in the positive ion mode.

K_2_EDTA blank mouse plasma purchased from Valley Biomedical, Winchester, VA, USA, was used to prepare standards and quality control (QC) samples for plasma quantitation and as surrogates for brain homogenate quantitation. Twelve-point calibration samples were prepared by spiking the corresponding analytes into blank mouse plasma, resulting in a concentration range from 2.34 ng/mL to 4800 ng/mL. QC samples were prepared at 14.0 ng/mL, 225 ng/mL, and 3600 ng/mL and analyzed in triplicate. Sample concentrations were determined using a linear calibration function, weighted 1/x or 1/x^2^, or a quadratic calibration function, weighted 1/x^2^, generated by the regression of the ratio of the peak area of the analyte to the peak area of its corresponding internal standard against the concentration of the analytes in the standard samples.

#### 4.4.3. Quantification of Fenfluramine, Norfenfluramine and Their l-Enantiomers in Brain

Bead mill tubes containing brain tissues were thawed at room temperature, and 2 mL of homogenization solvent (water: acetonitrile (1:1, *v*/*v*) was added. The tubes were placed in the Omni Bead Mill Homogenizer (Bead Ruptor Elite Model, Berlin, Germany) and were shaken at a velocity of 3.70 m/s for a single cycle lasting 30 s, with a second cycle if the homogenate was not uniform. The homogenized tubes were centrifuged at 4000 rpm for 20 min, and the supernatants were transferred to 1.5 mL Eppendorf tubes and stored frozen at −80 °C until analysis. The supernatants were assayed as described for supernatants obtained from plasma (Section 4.4.2), except that fenfluramine and its *l*-enantiomer were added to the calibrators and assayed together with metabolically formed norfenfluramine and *l*-norfenfluramine.

### 4.5. Assessment of Concentration-Response Relationships and Statistical Analysis

Concentration-response curves were generated using Microsoft Excel and GraphPad Prism version 9 software with data fitting according to the following Hill-Langmuir equation [42]:

E = B + (T − B) C^n^/(IC_50_^n^ + C^n^),

where:

E = response
B = bottom of the maximal effect (E_max_) set as 0.
T = top of the maximal effect (E_max_) set as 4.
C = concentration
N = the Hill coefficient or steepness of the concentration-response relationship
IC_50_ = concentration that reduces the response by half.

For plasma concentration-response curves, n values were derived directly from the data fitting. For brain tissue concentration-response curves, n was fixed (constrained) at −1.000.

All data are expressed as mean ± SD. Between-group differences were analyzed using one-way ANOVA with Dunnett’s post hoc test. Statistical significance was set at *p* < 0.05. All statistics were calculated using GraphPad Prism version 9.

## Figures and Tables

**Table 1:** Results of experiments to determine ED_50_ and TD_50_ values at the time of peak effect after intraperitoneal (i.p.) administration of *d*,*l*-fenfluramine, *d*,*l*-norfenfluramine, and their individual enantiomers in mice. In the maximal electroshock (MES) test, the time of peak effect was 4 h after dosing for *d*,*l*-norfenfluramine and *l*-norfenfluramine, 6 h after dosing for *d*,*l*-fenfluramine, *d*-fenfluramine, and *d*-norfenfluramine and 8 h after dosing for *l*-fenfluramine. For minimal motor impairment (MMI), the time of peak effect was 0.25 h after dosing for all compounds.

	Compounds and Doses Tested											
	*d*,*l*-Fenfluramine		*d*,*l*-Norfenfluramine		*d*-Fenfluramine		*l*-Fenfluramine		*d*-Norfenfluramine		*l*-Norfenfluramine	
Test	Dose (mg/kg)	N/T	Dose (mg/kg)	N/T	Dose (mg/kg)	N/T	Dose (mg/kg)	N/T	Dose (mg/kg)	N/T	Dose (mg/kg)	N/T
Maximal electro-shock (MES)	2.5	0/8 ^1^	1	0/8	2.5	0/8	4	2/8	0.25	0/8 ^1^	5	1/8
	5	3/8 ^2^	2.5	3/8	5	3/8	8	2/8 ^12^	1	0/8 ^1^	10	3/8
	10	4/8	5	3/8 ^5^	10	1/8	16	6/8	5	7/8	15	6/8
	15	7/8	10	6/8 ^6^	15	5/8	25	7/8	10	7/8	20	3/8
			20	4/8 ^6,7^	30	8/8	30	7/8	18	5/8	30	5/8
			30	8/8					25	7/8 ^12^	40	8/8
									30	6/8 ^2,19^		
Minimal motor impair-ment (MMI)	30	0/8	10	0/8	15	0/8	50	2/16 ^13^	2	1/8	5	0/8
	40	3/8	15	1/8	30	4/8	75	13/16 ^14,15^	5	4/8 ^20^	10	0/8
	50	5/8	20	5/8	45	6/8 ^8,9,10^	100	16/16 ^4,16,17,18^	10	6/8	20	3/8
	60	8/8 ^3,4^	25	4/8	60	8/8 ^4,11^			20	8/8 ^11,21,22^	25	6/8
			30	8/8					30	8/8 ^4,11^	50	8/8 ^4,14^
									45	8/8 ^11,21,23,24,25^		

*Note*: Any adverse effects are also noted. An observer blinded to treatment confirmed the behavioural observations. *Abbreviations*: N, number of animals protected (seizure activity tests) or number of animals displaying motor impairment; T, number of animals tested. ^1^ Death following tonic extension in four or ^2^ in two animals; ^3^ clonic seizures in four animals, ^4^ tremors in eight animals; ^5^ cold tails in six or ^6^ in five animals; ^7^ four mice soft to touch; ^8^ ataxia, ^9^ unable to grasp rotarod and ^10^ tremors in three animals; ^11^ unable to grasp rotarod in eight animals; ^12^ death following tonic extension in one animal; ^13^ tremors in nine animals; ^14^ unable to grasp rotarod in two animals; ^15^ tremors in four animals; ^16^ ataxia in eight animals; ^17^ unable to grasp rotarod in fourteen animals; ^18^ stretching and rolling in one animal; ^19^ death without tonic extension in one animal; ^20^ tremors and ^21^ severe tremors in two animals; ^22^ tremors in six animals; ^23^ loss of righting reflex in one animal; ^24^ one animal died during test without seizure; ^25^ tremors in five animals.

**Table 2:** Median effective dose (ED_50_) values for the antiseizure activity of *d*,*l*-fenfluramine, *d*,*l*-norfenfluramine and their individual enantiomers in the maximal electroshock seizure (MES) model in mice. Median toxic dose (TD_50_) and protective index (PI, TD_50_/ED_50_) values are also shown. For comparison purposes, the table also includes previously published ED_50_ values in the MES model, TD_50_ and PI values in rats [25]. All doses are expressed as the HCl salt.

	ED_50_ (mg/kg) *	TD_50_ (mg/kg) *	PI
Mice			
*d*,*l*-Fenfluramine	8.1 (5.3–12.4)	44.4 (38.5–49.8)	5.5
*d*,*l*-Norfenfluramine	7.0 (3.52–12.6)	20.7 (17.2–24.2)	2.9
*d*-Fenfluramine	11.4 (7.4–17.6)	32.3 (22.8–39.7)	2.8
*l*-Fenfluramine	10.0 (5.0–15.0)	62.7 (55.7–69.5)	6.3
*d*-Norfenfluramine	5.1 (2.1–8.8)	5.1 (2.9–7.5)	1.0
*l*-Norfenfluramine	14.8 (8.3–22.1) *	21.6 (10–50)	1.5
Rats			
*d*,*l*-Fenfluramine	10.7 (6.4–15.4)	27.8 (22.3–33)	2.6
*d*,*l*-Norfenfluramine	8.7 (7.2–10.4)	10–15 **	not assessed
*d*-Fenfluramine	8.4 (6.6–10.6)	26.7 (21.0–34.8)	3.2
*l*-Fenfluramine	13.4 (10.1–16.3)	38.5 (34.6–42)	2.9
*d*-Norfenfluramine	≈5 **	≈5 **	not assessed
*l*-Norfenfluramine	10.2 (6.8–13.0)	12.5 (8.7–16.1)	1.2

* Values in brackets are 95% confidence intervals (95%CI) determined using probit analysis. The 95%CI for. *l*-norfenfluramine ED_50_ in rats were not reported in the original publication and was calculated at a later time. ** Tentative estimate due to insufficient data.

**Table 3:** The antiseizure activity of *d*,*l*-fenfluramine, *d*,*l*-norfenfluramine and their *l*-enantiomers in the audiogenic seizure model in DBA/2 mice. The table also shows the concentration of the same compounds in plasma and brain tissue soon after seizure testing. Experiments 1 to 4 were conducted at the Canadian site, and experiments 5 and 6 at the French site (no plasma was collected at the French site). All doses are expressed as the HCl salt.

Experiment	Compound	Dose (mg/kg)	N. of Animals	Mean Seizure Score ± SD *	Mean Plasma Concentration ± SD (ng/mL)	Mean Brain Concentration ± SD (ng/g)	Brain/Plasma Concentration Ratio
1	Vehicle	N/A	8	4.00 ± 0.00	N/A	N/A	N/A
	*l*- Norfenfluramine	5	8	0.50 ± 0.54	442 ± 40.0	7780 ± 629	17.6
		10	8	0.38 ± 0.52	898 ± 62.5	16,900 ± 1680	18.7
		20	8	0.00 ± 0.00	1950 ± 297	34,400 ± 3790	17.6
2	Vehicle	N/A	8	3.38 ± 1.19	N/A	N/A	N/A
	*l*-Norfenfluramine	0.5	8	3.25 ± 1.39	43.5 ± 9.9	798 ± 91	18.4
		1	8	2.00 ± 1.77	83.7 ± 18.8	1770 ± 127	21.2
	*d*,*l*-Fenfluramine	15	8	0.13 ± 0.35	1080 ± 98.4 * (131 ± 18.7)	23,400 ± 1630 * (3016 ± 325)	21.6
3	Vehicle	N/A	7	2.57 ± 1.81	N/A	N/A	N/A
	*d*,*l*-Norfenfluramine	0.3	8	3.75 ± 0.71	22.6 ± 2.1	299 ± 24	13.2
		1	8	1.63 ± 0.52	60.3 ± 17.6	981 ± 130	16.3
		3	8	1.50 ± 0.54	184 ± 42.7	3450 ± 429	18.8
4	Vehicle	N/A	8	3.38 ± 0.74	N/A	N/A	N/A
	*d*,*l*-Norfenfluramine	10	8	0.63 ± 0.52	1090 ± 194	22,400 ± 4740	20.5
5	Vehicle	N/A	10	3.20 ± 0.42	N/A	N/A	N/A
	*d*,*l*-Fenfluramine	17.4	10	1.60 ± 0.52	N/A	21,300 ± 3760 * (2459 ± 437)	N/A
		34.7	10	0.90 ± 0.57	N/A	40,200 ± 6860 * (3094 ± 380)	N/A
	*l*-Fenfluramine	34.7	10	1.60 ± 0.52	N/A	52,200 ± 8550	N/A
						* (3036 ± 400)	
	Sodium valproate	180	10	0.40 ± 0.52	N/A	N/A	N/A
6	Vehicle	N/A	10	3.10 ± 1.20	N/A	N/A	N/A
	*l*-Fenfluramine	8.7	10	2.80 ± 1.03	N/A	9300 ± 2690 * (1274 ± 219)	N/A
		17.4	10	2.10 ± 0.74	N/A	19,700 ± 2470 * (1976 ± 459)	N/A

N/A, not assessed or not applicable. * *d*,*l*- and *l*-norfenfluramine concentration as a metabolite of *d*,*l*- and *l*-fenfluramine, respectively.

**Table 4:** Median effective doses (ED_50_) of *d*,*l*-fenfluramine, *d*,*l*-norfenfluramine and their *l*-enantiomers (expressed as the HCl salt) in the audiogenic seizure model in DBA/2 mice. Median effective concentrations (EC_50_) of the same compounds in plasma and brain are also shown. ED_50_ and EC_50_ values were calculated based on the results shown in Table 2.

Compound	ED_50_ (mg/kg)	EC_50_	
		Plasma (ng/mL)	Brain (ng/g)
*l*-Norfenfluramine	1.18	101	1940
*d*,*l*-Norfenfluramine	1.28	81	1350
*l*-Fenfluramine	20.5	N/A	25,400
*d*,*l*-Fenfluramine	11.8	N/A	13,200
*l*-Norfenfluramine (as metabolite of *l*-fenfluramine)	N/A	N/A	2330
*d*,*l*-Norfenfluramine (as metabolite of *d*,*l*-fenfluramine)	N/A	N/A	1270

N/A, not assessed or not applicable.

## Data Availability

The original contributions presented in the study are included in the article. Further inquiries can be directed to the corresponding author/s.
